# Reduced expression of small GTPases and hypermethylation of the folate binding protein gene in cisplatin-resistant cells

**DOI:** 10.1038/sj.bjc.6601956

**Published:** 2004-06-15

**Authors:** D-W Shen, A Su, X-J Liang, A Pai-Panandiker, M M Gottesman

**Affiliations:** 1 Laboratory of Cell Biology, Center for Cancer Research, National Cancer Institute, National Institutes of Health, 37 Convent Drive, Bethesda, MD 20892-4254

**Keywords:** small GTPases, endocytosis, FBP, DNA hypermethylation

## Abstract

Reduced accumulation of cisplatin is the most consistent feature seen in cisplatin-resistant (CP-r) cells that are cross-resistant to other cytotoxic compounds, such as methotrexate. In this report, defective uptake of a broad range of compounds, including [^14^C]-carboplatin, [^3^H]MTX, [^3^H]folic acid (FA), [^125^I]epidermal growth factor, ^59^Fe, [^3^H]glucose, and [^3^H]proline, as well as ^73^As^5+^ and ^73^As^3+^, was detected in CP-r human hepatoma and epidermal carcinoma cells that we have previously shown are defective in fluid-phase endocytosis. Downregulation of several small GTPases, such as rab5, rac1, and rhoA, which regulate endocytosis, was found in CP-r cells. However, expression of an early endosomal protein and clathrin heavy chain was not changed, suggesting that the defective endocytic pathway is clathrin independent. Reduced expression of the cell surface protein, folate-binding protein (FBP), which is a carrier for the uptake of MTX, was also observed in the CP-r cells by confocal immunofluorescence microscopy and Real-Time PCR. Reactivation of the silenced FBP gene in the CP-r cells by a DNA demethylation agent, 2-deoxy-5-aza-cytidine (DAC) demonstrates that hypermethylation occurred in the CP-r cells. The uptake of [^14^C]carboplatin, [^3^H]FA, and [^3^H]MTX increased in an early stage CP-r cell line (KB-CP1) after treatment with DAC. Both a defective endocytic pathway and DNA hypermethylation resulting in the downregulation of small regulatory GTPases and cell surface receptors contribute to the reduced accumulation of a broad range of compounds in CP-r cells.

Cisplatin (*cis*-diamminedichloroplatinum II) has become a major chemotherapeutic agent for treatment of a wide spectrum of solid tumours. Biochemical, cellular, and molecular approaches have been used to identify the molecular basis of resistance to cisplatin. Development of resistance to cisplatin *in vivo* and in cell lines is multifactorial, including changes in DNA repair proficiency (Fink *et al*, 1998; Branch *et al*, 2000; Fojo, 2001), proteins and enzymes involved in detoxicifications of cisplatin, such as metallothionein and glutathione-related enzymes (Kelly *et al*, 1988; Godwin *et al*, 1992; Daubeuf *et al*, 2002), chaperones (Yamamoto *et al*, 2001), signal transduction pathways (Basu *et al*, 1996), and cell cycle regulators (Kondo *et al*, 2001). Alterations in the expression of proto-oncogenes, apoptosis-related genes, and cancer susceptibility genes have also been described in association with cisplatin resistance (Lowe *et al*, 1993; Husain *et al*, 1998; Slupianek *et al*, 2001). Recently, molecular approaches using cDNA microarrays and differential display have provided useful tools for further understanding of the molecular mechanisms that contribute to cisplatin resistance (Grottke *et al*, 2000; Niedner *et al*, 2001).

The reduced accumulation of cisplatin is the most consistent feature seen in cisplatin-resistant (CP-r) cells (Loh *et al*, 1992; Johnson *et al*, 1996; Shen *et al*, 2000). Mechanisms for drug resistance resulting from the reduced accumulation of drugs have been categorised as decreased influx (impaired uptake) and increased efflux (active export) (Gottesman *et al*, 2002). We have previously reported that a pleiotropic defect resulting in the reduced accumulation of [^3^H]MTX, ^73^As^5+^, and ^73^As^5+^ in the CP-r cells was found to be associated with reduced expression of folate-binding protein (FBP) and arsenic-binding proteins (Shen *et al*, 1998). The reduced accumulation of [^14^C]carboplatin in CP-r cells in association with crossresistance to this compound was also documented to be due to a defect in energy-dependent uptake and not due to active efflux (Shen *et al*, 2000). However, the cell biology and molecular bases of the reduced accumulation in CP-r cells need to be further elucidated.

A defect in fluid-phase endocytosis was found in higher level CP-r cell lines isolated in multiple steps (Chauhan *et al*, 2003). To investigate the cellular and molecular genetic events involved in cisplatin resistance, particularly for the reduced uptake of a variety of compounds, reduced expression of several small GTPases involved in the endocytic pathway and FBP was found in the CP-r cells as detected by confocal immunofluorescence microscopy, immunoblotting, and realtime (RT)-PCR. Reactivation of the silenced FBP gene in the CP-r cells by a demethylation agent, 2-deoxy, 5-aza-cytidine (DAC), indicated that hypermethylation of the gene occurred during the development of cisplatin resistance, and that the uptake defect could be reversed to some extent by DAC. The data presented here provide evidence that CP-r cells have a global defect in the uptake of cisplatin and several other related and unrelated compounds at least related in part to reduced expression of small GTPases that regulate in endocytic pathways and DNA hypermethylation in the CP-r cells may play a role in this pleiotropic phenotype.

## MATERIALS AND METHODS

### Cell lines and cell culture

Two populations of CP-r cell lines and their parental cell lines were studied: the human epidermoid carcinoma cell line KB-3-1 and its CP-r derivative, KB-CP20, and the human liver carcinoma cell line BEL-7404 and its CP-r derivative, 7404-CP20.

**Figure 1:**
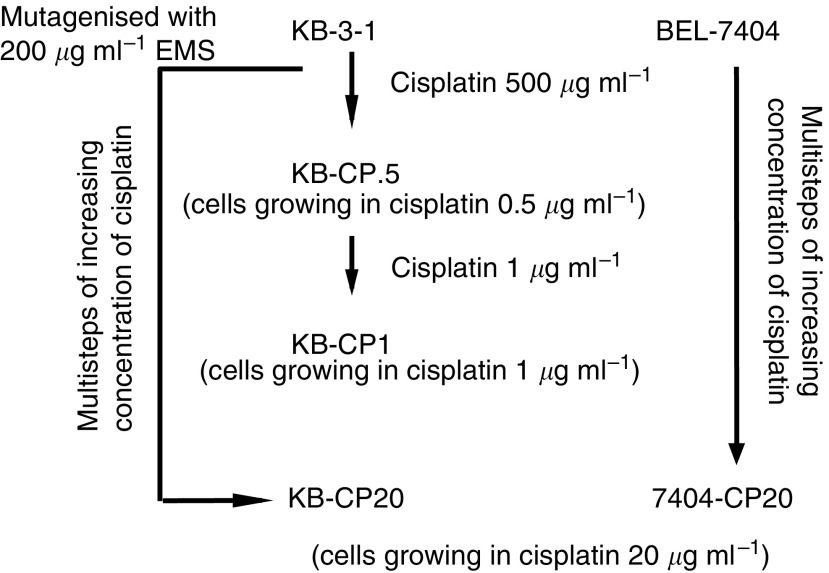
A flow diagram showing the derivation of CP-r cell lines isolated from two-step and multistep selection with cisplatin from KB-3-1 (human epidermoid carcinoma cells) and BEL-7404 (human liver carcinoma cells).

Both human CP-r cell lines were selected in 20 *μ*g cisplatin ml^−1^ medium as described previously (Shen *et al*, 1998). The early-stage CP-r cell line KB-CP1 was isolated from the KB-3-1 cells in a two-step selection including 0.3 and 1 *μ*g ml^−1^ cisplatin (Liang *et al*, 2003). Figure 1

shows a flow diagram of the derivation of CP-r cell lines isolated from KB-3-1 and BEL-7404 cells that were used in this study. All cell lines were grown as monolayer cultures at 37°C in 5% CO_2_, using Dulbecco's modified Eagle's medium with 4.5 g l^−1^ glucose (Invitrogen, Grand Island, NY, USA), supplemented with L-glutamine, penicillin, streptomycin, and 10% foetal bovine serum (BioWhittaker, Walkersville, MD, USA).

### Drugs and radiochemicals

All chemicals were purchased from Sigma (St Louis, MO, USA) unless otherwise noted. Radioactive labelled compounds, such as [^14^C]carboplatin, [^3^H]MTX, [^3^H]folic acid (FA), [^125^I]epidermal growth factor (EGF), and ^59^Fe, were purchased from Amersham Biosciences (Piscataway, NJ, USA). Arsenic-73 at specific activity 242 Ci mmol^−1^ was provided by the Los Alamos National Laboratory. ^73^As^5+^ was prepared by the reduction of ^73^As^5+^ 50 *μ*l of reduction solution consisting of 0.1 mM NaAsO2, 66 mM Na_2_S_2_O_5_, 27 mM Na_2_S_2_O_3_, and 82 mM H_2_SO_4_ was added to 50 *μ*l of 50 *μ*Ci of ^73^As^5+^ (H_3_^73^AsO_4_) and incubated for 40 min at room temperature according to Reay and Asher (1997).

### Measurement of the accumulation of radiochemicals

Dishes containing a subconfluent monolayer of cells were used for the assay. For the uptake analysis of [^3^H]FA and [^3^H]MTX, cells were washed once with FA-deficient DMEM (Invitrogen), then incubated at 37°C with the same medium containing 0.5 *μ*Ci ml^−1^ of radioactive materials. For all other radioactive compounds, cells were incubated with the regular medium containing 0.5 *μ*Ci ml^−1^ of radioactive materials. After a 1- or 2-h incubation, uptake was stopped by washing the dishes three times with ice-cold phosphate-buffered saline, then harvesting cells by trypsinisation. The cell suspensions were transferred from the dishes into counting vials with cocktail Ecoscint A (National Diagnostics, Atlanta, GA, USA) and counted in a Beckman LS3801 liquid scintillation counter. The radioactivity of the cisplatin-sensitive (CP-s) parental KB-3-1 cells incubated with various radioactive compounds was calculated as 100% accumulation, in comparison to the KB-CP20 cells. Duplicate dishes of cells for each determination were analysed.

### Preparation of enriched plasma membrane proteins

Membrane proteins were purified according to the method of Cornwell *et al* (1987). Briefly, 1 × 10^9^ cells from each cell line were disrupted on ice by nitrogen cavitation with constant stirring for 40 min. Two cycles of sucrose gradient ultracentrifugation were followed. The purified membrane pellets were resuspended in 3 ml TSNa buffer (10 mM Tris-HCl pH 7.45, 250 mM sucrose, 50 mM NaCl, and 1% aprotinin) and stored at −80°C until use.

### Immunoblotting

Sodium dodecyl sulphate–polyacrylamide gel electrophoresis (SDS–PAGE) immunoblotting was carried out as described previously (Shen *et al*, 1998). Minigels were run as recommended by the manufacturer (Invitrogen). Following electrophoresis, the gels were transblotted onto nitrocellulose membranes (Schleicher & Schuell, Keene, NH, USA) at 4°C. Immunoreaction was performed with desired primary antibodies and secondary HRP-conjugated antibodies. Pierce ECL reagents (Pierce Biotechnology, Rockford, IL, USA) were used for developing signals as described by the manufacturer. Specific first antibodies and HRP-labelled secondary antibodies were purchased from BD Biosciences (San Diego, CA, USA) and Jackson Immuno-Research Lab (West Grove, PA, USA), respectively, except as indicated.

### Confocal image analysis

Cells were cultured in a Lab-Tek Chamber Slide (Nalge Nunc International, Naperville, IL, USA) and fixed with 70% ETOH at −20°C for 15 min. The fixed cells were reacted with the primary antibodies and then followed by rhodamine-labelled anti-mouse IgG second antibody. FBP-specific monoclonal antibody MOv19 was a gift from MI Colnagh (Mantovani *et al*, 1994). Immunofluorescent images of cells were monitored under a laser scanning confocal microscope (Bio-Rad) at × 600 magnification.

### RT-PCR

RNA was extracted by Qiagen RNeasy kits as described by the manufacturer (Qiagen, Valencia, CA, USA). RT-PCR on the LightCycler instrument (Roche Diagnostics, Indianapolis, IN, USA) was performed in a total volume of 20 *μ*l in the presence of 2 *μ*l of RNA (0.5 *μ*g) or water as control. The resolution solution (Roche, STBR Green I kit) was applied with 2 *μ*l per reaction for a better resolution of melting curves and a significant increase in sensitivity as for GC-rich templates. Specific primers for desired genes were added to a final concentration of 10 pmol of each oligonucleotide primer. Quantitative analysis of the cDNA products was determined by the crossing point after 35 cycles of amplification. A DNA agarose gel was performed to check the size of the cDNA products and the intensity of fluorescence.

## RESULTS

### Reduced accumulation of radioactive compounds

**Figure 2:**
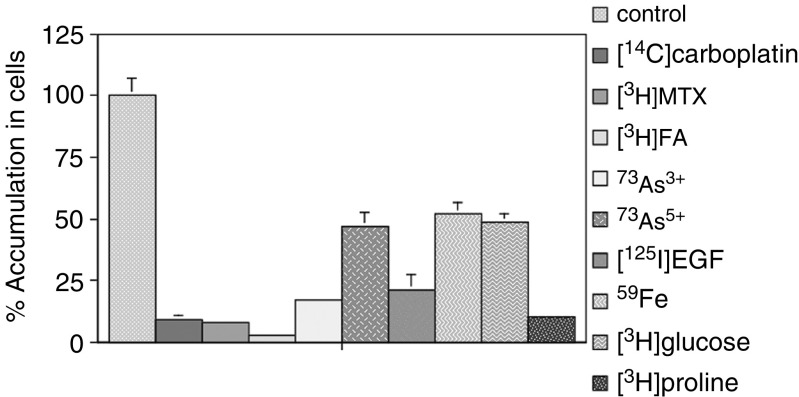
Uptake assay for various radioactively labelled compounds in KB-CP20 cells in comparison to parental CP-s KB-3-1 cells. The accumulation rate in the CP-s was taken as 100%. Cell lines used are described in Figure 1. Cells were incubated with the indicated radioactive compound at 37°C for 1 h. Measurement of radioactivity i-n cells is described in Materials and methods.

In our previous reports, decreased accumulation of [^14^C]carboplatin, [^3^H]MTX, ^73^As^3+^, and ^73^As^5+^ was found in the CP-r cells. In this report, we demonstrate that the KB-CP20 cells have a more global defect in the uptake of a variety of radioactively labelled compounds. As shown in Figure 2

, the accumulation of [^14^C]carboplatin, [^3^H]MTX, and [^3^H]FA (FA) was decreased over 90% in the CP-r cells compared with the CP-s cells. The decreased accumulation of [^3^H]glucose and [^3^H]proline was detected in the CP-r cells. The uptake of ^73^As^3+^, ^73^As^5+^, ^59^Fe, and [^125^I]EGF was also decreased from 52% to over 80% in the CP-r cells as compared to CP-s cells. The reduced accumulation of [^125^I]EGF is correlated with decreased expression of the epidermal growth factor receptor (EGF-R) in our CP-r cells as described previously (Chauhan *et al*, 2003).

### Reduced expression of small GTPases

**Figure 3:**
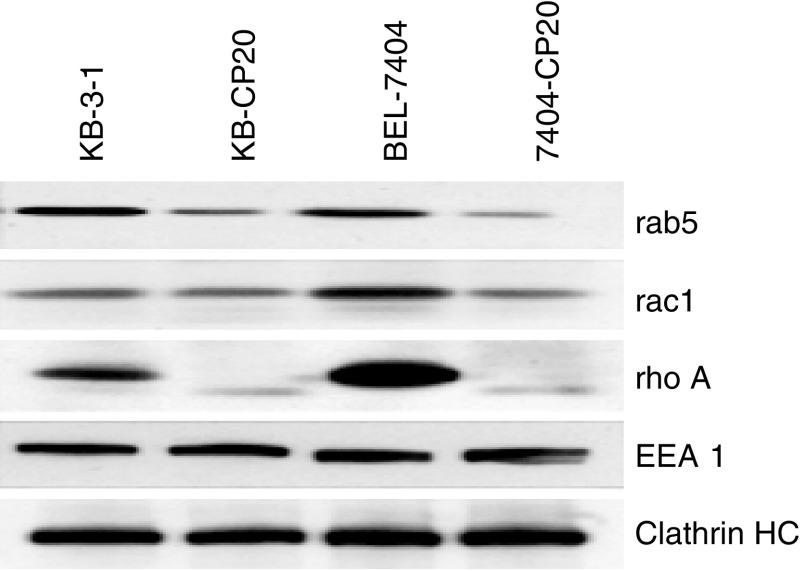
Protein profiles by immunoblot analysis as visualised by ECL. Enriched plasma membranes were transblotted onto nitrocellulose membranes after 10% SDS–PAGE as described in Materials and methods. Cells lines used in this analysis are described in Figure 1.

We have found that KB-CP20 cells have a defect in nonreceptor-mediated endocytosis (Chauhan *et al*, 2003) and wondered if this endocytosis defect in CP-r cells might account for some of the reduced uptake of nutrients and cytotoxic compounds in CP-r cells. To explore the endocytosis pathway further, we checked whether small GTPases and other related proteins known to regulate endocytosis might also be affected. By immunoblotting analysis on small GTPases, the expression levels of rab5 and rac1 were noticeably decreased, while rhoA was almost undetectable in both KB-CP20 and 7404-CP20 cells in comparison to their parental CP-s cell lines (Figure 3

). Quantitative RT-PCR analysis of mRNA for these small GTPases showed only modest decreases in mRNA levels (Table 1

**Table 1:** Reduced expression of several small GTPases and cell surface proteins in CP-r cells, measured by RT-PCR^a^

	**Levels of expression^a^**	
**Genes**	**KB-3-1**	**KB-CP20**
rab5	1	0.8
rac1	1	0.5
rhoA	1	0.4
FBP	1	<0.0015
RFC	1	0.6
DHFR	1	0.63

RT-PCR=real-time PCR; FBP=folate-binding protein; RFC=reduced folate carrier; DHFR=dehydrofolate reductase.

a Levels of gene expression are based on the differences in crossing points, which represents the number of gene duplication cycles as determined by a LightCycler RT-PCR.

), indicating that post-transcriptional regulation might also be involved in the reduction in protein levels. The expression levels of EEA1, an early endosome marker, and a clathrin-heavy chain that is involved in receptor-mediated endocytosis, were similar in these two pairs of human CP-r and CP-s cell lines, indicating that the reduced accumulation of a variety of radioactive compounds is not associated with changes of these two intracellular components. Expression of caveolin was increased significantly in both KB-CP20 and 7404-CP20 (data not shown).

### Reactivation of silenced FBP genes by a DNA methylation inhibitor

**Figure 4:**
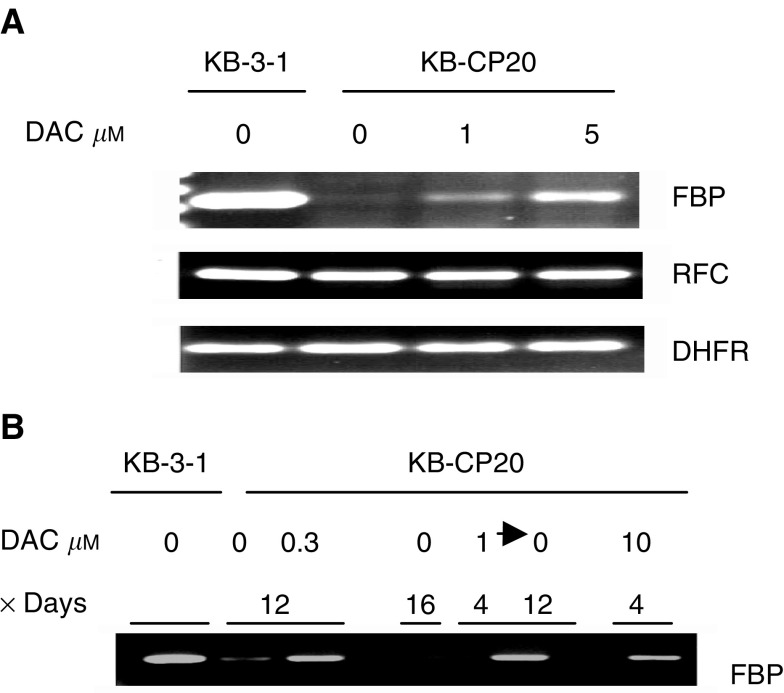
Reverse transcriptase PCR analysis on gene expression. (**A**) FBP, RFC, and DHFR genes. (**B**) Duration of activation of FBP gene by DAC, and the concentrations and time periods of DAC treatment were marked in the figure. RNA preparation and RT-PCR were performed as described in Materials and methods. KB-3-1 and KB-CP20 cells were described in Figure 1.

Since mRNAs encoding many different proteins were reduced in amount in CP-r cells, we sought to determine whether DNA hypermethylation might account for decreased transcription of certain genes in CP-r cells. In our previous paper, we reported that FBP and its mRNA were almost absent in the CP-r cells by Northern and immunoblot analysis (Shen *et al*, 1998). To study further the mechanism by which the FBP gene was downregulated, DAC, a DNA demethylation agent, was used to determine whether the silenced FBP gene could be reactivated as would be expected if the reduced expression of FBP in CP-r cells were due to hypermethylation of the gene. The expression levels of the FBP gene in CP-r cells were significantly elevated after exposure to DAC at 10 *μ*M for 3 days (Figure 4A

, upper panel). There were no changes in CP-s cells after treatment with DAC (data not shown), indicating that DAC has a specific effect on the activation of silenced FBP gene in CP-r cells.

There are at least three genes that are known to be commonly involved in MTX resistance: FBP, reduced folate carrier (RFC), and dehydrofolate reductase (DHFR) (Hsueh and Dolnick, 1994; Kelland *et al*, 1995; Moscow *et al*, 1995). Figure 4A shows clearly that only the FBP gene was silenced in the KB-CP20 cells, whereas changes in the expression of RFC and DHFR genes were undetectable. After treatment with DAC, expression levels of the FBP gene were elevated as the concentrations of DAC increased. There was no effect on the expression of RFC and DHFR by DAC treatment. These results demonstrate that the FBP gene was specifically downregulated during the acquisition of cisplatin resistance and could be reactivated by demethylation of the gene with DAC.

How persistent was DAC-activated gene expression? Figure 4B demonstrates that the expression of the FBP gene induced by DAC persisted for at least 12 days after initial exposure to the methylation inhibitor for 4 days. The induced expression levels of the FBP gene were quite similar among the CP-r cells treated with DAC at 0.3 *μ*M for 12 days, or 10 *μ*M for 4 days, or 1 *μ*M for 4 days followed by DAC-free medium for 12 days.

### Increased uptake of ^14^C-carboplatin, [^3^H]FA and [^3^H]MTX induced by DAC

**Figure 5:**
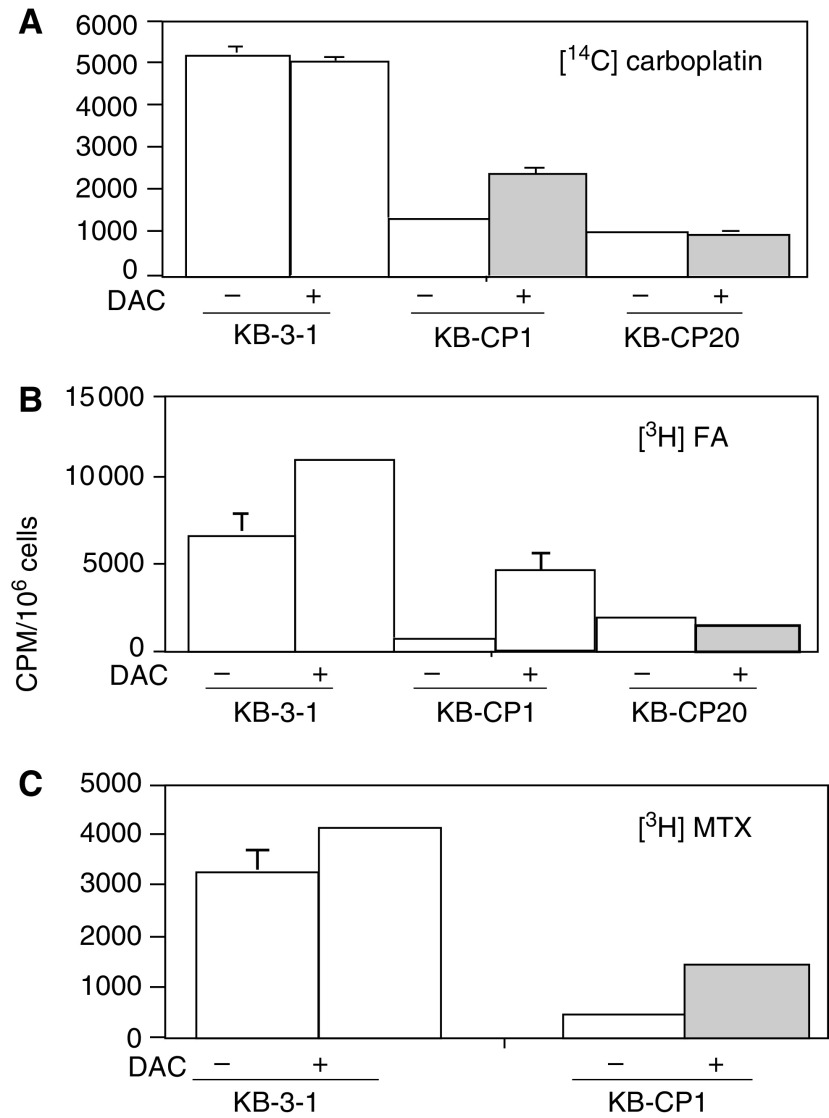
Uptake assay for radioactively labelled compounds in the CP-s and CP-r cells after treatment with DAC. Cells were incubated with the desired radioactive compound at 37°C for 2 h. (**A**) [^14^C]carboplatin, (**B**) [^3^H]FA, and (**C**) [^3^H]MTX. KB-3-1 and KB-CP20 cells were described in Figure 1.

**Figure 6:**
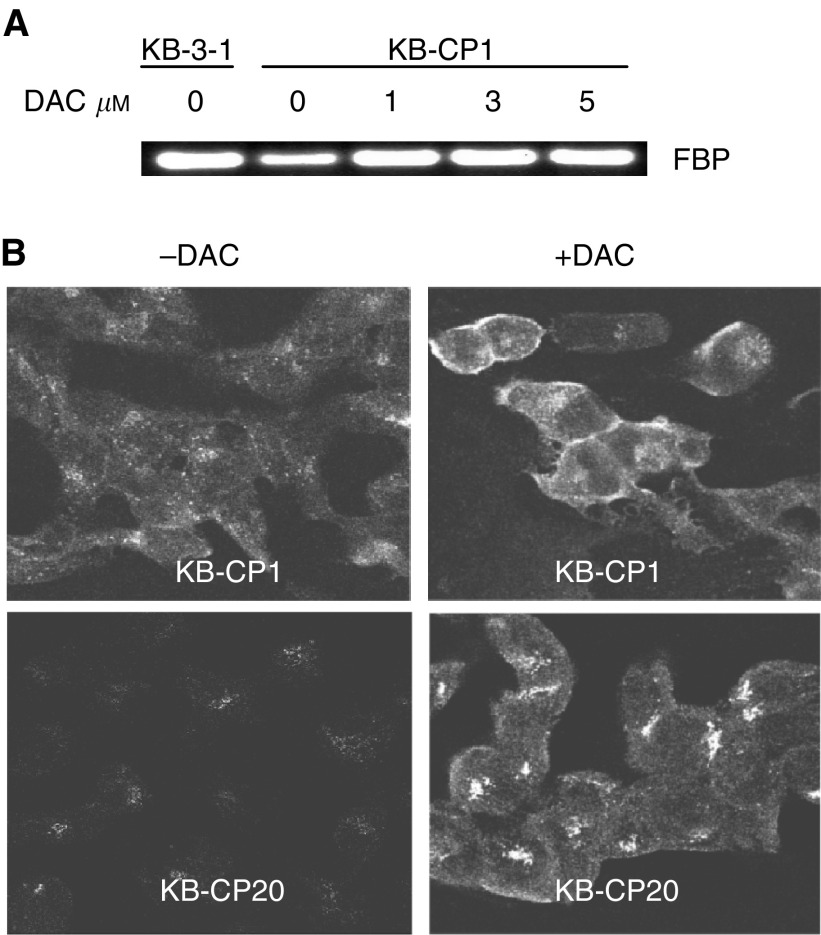
(**A**) Reverse transcriptase PCR analysis of DAC activation on KB-CP1 cells. The cell lines used in this study were described in Figure 1. Measurement of radioactivity in cells is described in Materials and methods. (**B**) Confocal microscopic immunofluorescent images of FBP stained by a monoclonal antibody specific to human FBP. Immunofluorescent images of cells were monitored under a laser scanning confocal microscope (Bio-Rad) at × 600 magnification.

Since reduced expression of the FBP gene in CP-r cells could be reactivated by DAC as seen in Figure 3A, we asked whether induced gene expression in CP-r cells would result in increasing the uptake of compounds whose uptake was reduced in the resistant cells. Uptake assays were performed using radioactively labelled compounds, [^14^C]carboplatin, [^3^H]FA, and [^3^H]MTX. Cells were treated with DAC, 1 *μ*M for KB-3-1 and KB-CP1 cells, and 5 *μ*M for KB-CP20 cells at 37°C for 3 days, then reincubated with DAC-free medium for another 3 days for recovery. After incubation with the desired radioactively labelled compounds at 37°C for 2 h, increased accumulation of [^14^C]carboplatin could be seen in DAC-treated two-step selected KB-CP1 cells only (Figure 5A

). There was little effect of DAC on the parental KB-3-1 cell line or on the KB-CP20 cell line. The uptake of [^3^H]FA and [^3^H]MTX could be stimulated up to 687 and 398%, respectively, in DAC-treated KB-CP1 cells compared to untreated cells (Figure 5B and C). For FA and methotrexate uptake, DAC also had less than a two-fold effect on the parental KB-3-1 cells. However, the KB-CP20 cells were nonresponsive to DAC treatment in the accumulation of [^3^H]FA. In parallel with the increased uptake of the above radioactively labelled compounds, the expression of the FBP gene in KB-CP1 cells was also increased after treatment with DAC for 3 days and then 3 days in DAC-free medium as detected by RT-PCR (Figure 6A

). Immunostaining with a monoclonal antibody specific to FBP detected by confocal microscopy indicated that the intensity of the fluorescence was increased in both DAC-treated KB-CP1 and KB-CP20 cells in comparison with their untreated control cells (Figure 6B). FBP was largely localised at the cell surface in the KB-CP1 cells after DAC activation, while the protein was mostly found in a Golgi-like distribution inside the cell in the KB-CP20 cells treated with DAC, explaining why this increased expression did not result in increased uptake of methotrexate and FA.

## DISCUSSION

In this work, we have examined the cellular and molecular basis of an extremely pleiotropic phenotype of CP-r cells that creates global alterations in gene expression resulting in altered expression of cell surface protein and decreased endocytosis. These changes have the net effect of decreased accumulation of cisplatin and many other compounds. Unlike other forms of multidrug resistance due to energy-dependent efflux pumps, such as MDR1, MXR (ABCG2), and MRP family members (Borst and Elferink, 2002; Gottesman *et al*, 2002), this form of multidrug resistance causes decreased drug accumulation by blocking normal modes by which agents such as cisplatin enter cells. In this work, we show an association between the reduced uptake of a variety of cisplatin-related or -unrelated radioactive compounds and a defective endocytic pathway that could be reactivated by the DNA methylation inhibitor DAC.

Recently, decreased intracellular accumulation of cisplatin, one of the most important characteristics of CP-r cells, has attracted investigators' interest. The mechanism(s) by which cisplatin enters the cell and how decreased accumulation occurs in resistant cells are still not defined. Generally, the pathways by which cisplatin and its analogs are thought to enter the cell can be subdivided into three categories: passive diffusion, carrier or receptor-mediated transport, and entry via ion channels (Gottesman *et al*, 2002). Our previous data demonstrated that [^14^C]carboplatin enters cells via an energy-dependent transport process (Shen *et al*, 2000), and the reduced accumulation of [^14^C]carboplatin was due to impaired influx and not due to active efflux. It has been found recently in our laboratory that cisplatin resistance is associated with a defect in endocytosis and changes in lysosomal acidification (Chauhan *et al*, 2003), with mislocalisation of transporters and carriers, such as MRP1 and FBP (Liang *et al*, 2003).

In this study, we sought to understand the molecular and biochemical basis of the defective endocytosis in the CP-r cells. The expression of small GTPases, such as rab5, rac1, and rhoA, which are regulators of the endocytosis machinery (Ellis and Mellor, 2000; Sonnichsen *et al*, 2000; Sorkin, 2000), was downregulated in the CP-r cells. However, clathrin, and the early endosomal marker EEA1, remained unchanged in the CP-r cells (Figure 3).

The above results indicate that impaired uptake of cisplatin, [^14^C]carboplatin, [^3^H]MTX, and other compounds in CP-r cells was associated with the downregulation of several small GTPases involved in regulating the endocytosis machinery, possibly also resulting in mislocalisation of transporters, receptors/carriers, such as FBP, EGFR, and TFR. The reduced uptake of dextran-10 and horseradish peroxidase, which are fluid-phase endocytosis markers, was observed in CP-r cells (Chauhan *et al*, 2003). Clathrin levels were relatively unaffected in our CP-r cells, indicating that the defective endocytic pathway in CP-r cells is clathrin independent. Although a decline in protein levels was not always directly correlated with reduced mRNA levels (Table 1 *vs* Figure 3), suggesting post-transcriptional regulation in some cases, reduced protein was always associated with some reduced mRNA.

The data presented in this work raise the question as to whether reduced transcription could account for the reduced expression of transporters, receptors, carriers, and small GTPases in the CP-r cells. DNA hypermethylation has been reported in multidrug-resistant cell lines, including CP-r cells (Plumb *et al*, 2000; Efferth *et al*, 2001; Borst and Elferink, 2002). A link between mismatch repair deficiency and cytotoxic drug resistance was ascribed to hypermethylation of the promoter regions of genes involved in DNA repair (Strathdee *et al*, 1999).

In our work, the FBP gene was chosen as a model because of its association with reduced uptake and crossresistance to MTX in CP-r cells. Preliminary results have shown differences in methylation patterns of the FBP gene in parental and CP-r cells (data not shown). To study the relation between the downregulation of gene expression and DNA hypermethylation, DAC, a demethylating agent, was applied to see if the silenced FBP gene and other genes could be reactivated. DAC has been shown to replace cytidine residues in replicating DNA and prevent methylation, thereby demethylating or inducing hypomethylation of DNA (Hsueh and Dolnick, 1994). It is evident from this work that the silenced FBP gene in KB-CP20 cells could be reactivated by DAC (Figure 4). The duration of reexpression of the FBP gene was at least 12 days after initial exposure to this compound for 4 days.

FBP, as mentioned above, and RFC are both involved in the uptake of MTX, while DHFR is a cytotoxic target for MTX. The data reported here indicate that only FBP expression was remarkably reduced and that there was little change in the expression of RFC and DHFR genes in the CP-r cells (Figure 4A). No response of the RFC gene to DAC treatment was observed, whereas the expression of DHFR was elevated about 2–3-fold after DAC induction.

Uptake assays on [^14^C]carboplatin, [^3^H]FA, and [^3^H]MTX in DAC-treated or -untreated cells revealed that DAC treatment increased the accumulation of these compounds in KB-CP1 cells. The parental KB-3-1 cells had no response to DAC for uptake of [^14^C]carboplatin with some increase in the uptake of [^3^H]FA and [^3^H]MTX. In contrast, DAC showed little effect on uptake in the high-level Cp-r KB-CP20 cells. Why did expression of the FBP gene in the KB-CP20 cells after treatment with DAC not result in increased uptake of [^3^H]FA and [^3^H]MTX? Confocal microscopic images revealed that expression of the FBP gene was elevated in the KB-CP20 cells, but FBP was not localised at the cell surface. Instead, it was distributed in the cytoplasm. Therefore, it could not function as a cell surface carrier for MTX. In the KB-CP1 cells, FBP induced by DAC was largely localised at the cell surface, explaining the increased uptake of MTX and FA under these conditions.

Taken together, these data suggest the existence of a novel cellular defense system or systems that result in cisplatin resistance and crossresistance to other related and unrelated drugs. Defects in this system, which cause decreased uptake of many compounds, lead to reduced expression and mislocalisation of certain genes, such as transporters, that is, MRP1 (Liang *et al*, 2003) and MRP2-4 (data not shown), carriers, that is, FBP, and genes involved in endocytic pathways and protein trafficking, that is, some small GTPases. In this work, we show that the FBP gene was turned down or off by DNA hypermethylation triggered by selection in cisplatin. Whether DNA methylation is a primary or secondary event is not clear, but demethylation by DAC results in at least partial re-expression and function of some of the gene products. These findings may provide valuable information for the design of regimens for cancer chemotherapy as well as improving the ability to detect drug resistance before and after chemotherapy with cisplatin by monitoring changes of DNA methylation status and expression levels of small GTPases and FBP in clinical tumour specimens.

**Figure 7:**
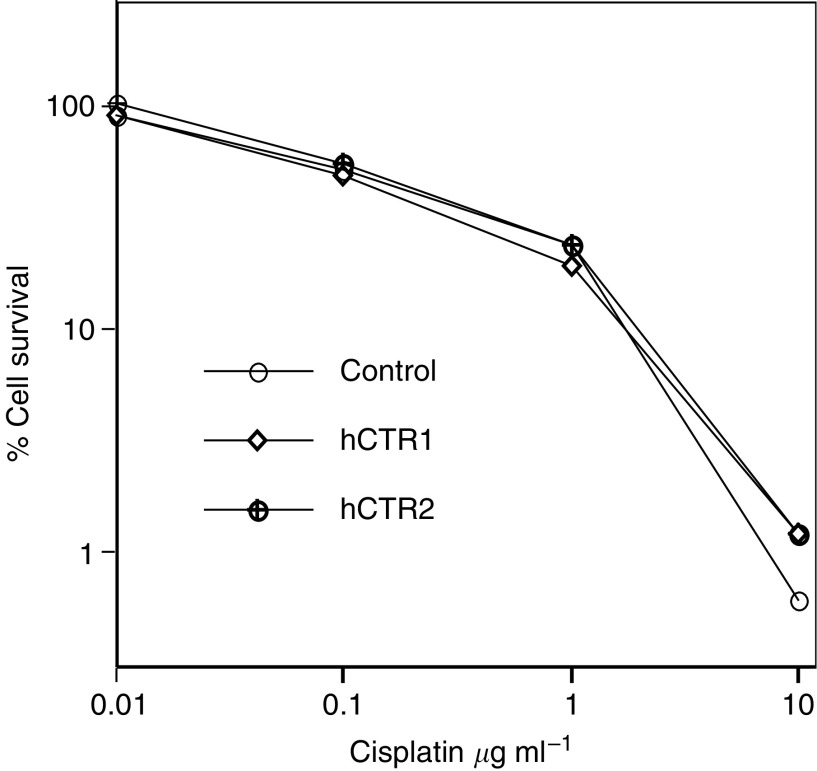
Cell killing curves show that there was little difference in cisplatin resistance between two individual stable transfected KB-3-1 cell lines with hCTR1/pcDNA3 and cells transfected with the empty vector in a 3-day assay as described in a previous study (Shen *et al*, 2000). KB-3-1 cells were described in Figure 1.

In an effort to see if cisplatin resistance is related to the overexpression of the copper transporter, we inserted the human copper transporter h*CTR1* (Zhou and Gitschier, 1997) into the expression vector pcDNA3.1, then transfected this into CP-s cells. Figure 7

shows killing curves indicating little difference in cells transfected with the control mock vector and two individual h*CTR1* expression vectors in levels of resistance to cisplatin.

Recent data from Howell's lab demonstrated that two P-type of ATPases ATP7A and ATP7B are involved in cisplatin resistance in human ovarian carcinoma cells by modulating cellular pharmacology of cisplatin and other metals, suggesting that a sequestration and secretory system may exist (Katano *et al*, 2002, 2003). Therefore, it is possible that there might be at least two pathways for the reduced accumulation of cisplatin and other related chemicals in CP-r cells: impaired uptake and active secretion. These results will help us to understand the multifactorial mechanisms in acquisition and development of cisplatin resistance in human cancer cells.
